# Phenolic Profiles and Antioxidant Activity of Lotus Root Varieties

**DOI:** 10.3390/molecules21070863

**Published:** 2016-06-30

**Authors:** Yang Yi, Jie Sun, Jun Xie, Ting Min, Li-Mei Wang, Hong-Xun Wang

**Affiliations:** 1 College of Food Science & Engineering, Wuhan Polytechnic University, Wuhan 430023, China; yiy86@whpu.edu.cn (Y.Y.); qiqijiayuguan@163.com (J.S.); 15927583334@163.com (J.X.); minting1323@163.com (T.M.); 2 Hubei Engineering Research Center for Fresh Food, Wuhan 430023, China; wanglimeiyx@163.com; 3 College of Biology and Pharmaceutical Engineering, Wuhan Polytechnic University, Wuhan 430023, China

**Keywords:** lotus root, variety, phenolic compound, antioxidant activity

## Abstract

Lotus root attracts increasing attention mainly because of its phenolic compounds known as natural antioxidants. Its thirteen varieties were systematically analyzed on the content, distribution, composition and antioxidant activity of phenolic compounds for a better understanding of this aquatic vegetable. The respective mean contents of total phenolics in their flesh, peel and nodes were 1.81, 4.30 and 7.35 mg gallic acid equivalents (GAE)/g fresh weight (FW), and those of total flavonoids were 3.35, 7.69 and 15.58 mg rutin equivalents/g FW. The phenolic composition determined by a high-performance liquid chromatography method varied significantly among varieties and parts. The phenolics of flesh were mainly composed of gallocatechin and catechin; those of peel and node were mainly composed of gallocatechin, gallic acid, catechin and epicatechin. The antioxidant activities of phenolic extracts in increasing order were flesh, peel and node; their mean concentrations for 50% inhibition of 2,2-diphenyl-1-picrylhydrazyl radical were 46.00, 26.43 and 21.72 µg GAE/mL, and their mean values representing ferric reducing antioxidant power were 75.91, 87.66 and 100.43 µg Trolox equivalents/100 µg GAE, respectively. “Zoumayang”, “Baheou”, “No. 5 elian” and “Guixi Fuou” were the hierarchically clustered varieties with relatively higher phenolic content and stronger antioxidant activity as compared with the others. Especially, their nodes and peels are promising sources of antioxidants for human nutrition.

## 1. Introduction

Lotus (*Nelumbo* *nucifera* Gaertn.), a well-known aquatic plant of family *Nelumbonaceae*, has been widely cultivated for food production, ornamental horticulture and traditional Asian medicine in China, Korea, Japan and India [1,2]. Its root, which contains abundant dietary fibers, starches, sugars, proteins, amino acids, minerals, vitamins and phenolics [3,4,5,6], is popularly consumed as both a delicious and nutritional vegetable and a therapeutic herb [1,7]. Some pharmacological potentials of the ethanol- and methanol-soluble extracts of lotus root, including antioxidant [2], immunomodulatory [8], antiobesity [2,7], hypoglycemic [9], psychopharmacological [10] and memory-improving activities [11], are proposed to be closely related to phenolic compounds. To our knowledge, these compounds in lotus root have attracted increasing attention mostly for the substrate of enzymatic browning [12,13,14], but not the bioactive components for human nutrition.

Phenolic compounds are secondary metabolites involved in several plant growth and development processes. As natural antioxidants, they have been extensively studied in recent years for lowering the risk of diseases associated with oxidative stress [15,16]. Among more than thirty-six selected vegetables, lotus root exhibited the strongest antioxidant activities [6,17]. Phenolic compounds were regarded as the main contributors in consideration of the significant positive correlation between phenolics content and antioxidant capacity [1,6,18]. The content and composition of phenolic compounds have been well investigated in various lotus tissues [19,20,21,22,23], except the root. The structurally characterized phenolic compounds of lotus root include catechol, gallic acid, (+)-catechin, (−)-epicatechin, (+/−)-gallocatechin and chlorogenic acid [5,14,24,25], but their contents, distributions and antioxidant activities remain unclear.

For most plants, the content, composition, and antioxidant activity of phenolic compounds vary significantly among their varieties, such as *Prunus* *persica* L. Batsch [26] and *Litchi* *chinensis* Sonn [27]. However, little is known about the varieties of lotus root. Peel and nodes are the major by-products of fresh-cut lotus root, which is a minimally processed food with increasing attention [12,13]. Accordingly, peel and nodes are gradually being focused on for utilization. Their higher phenolics contents and stronger antioxidant activities compared with flesh imply better development prospects [6,25].

Considering the growing number of lotus root-derived extracts/foodstuffs and the increasing literature on their biological properties, the detailed characterization of the phenolic compounds from lotus root, with regards to the three parts from different varieties, should be investigated to obtain valuable evidence that supports future efforts toward the development and utilization of this aquatic vegetable. Therefore, the objectives of this work were: (1) to compare the content of total phenolics and total flavonoids (TPC and TFC) in the flesh, peel and node of thirteen lotus root varieties; (2) to investigate the varietal differences of phytochemicals and antioxidant activity and their potential correlations; and (3) to indicate the preponderant varieties for the development of natural phenolics.

## 2. Results and Discussion

### 2.1. Contents of Total Phenolics and Total Flavonoids in the Different Parts of Lotus Root Varieties

As seen in Table 1, both total phenolics contents (TPC) and total flavonoids contents (TFC) differed significantly among the three parts of lotus root (*p* < 0.05), and could be ordered as node > peel >flesh, as reported in previous studies [1,6,25]. TPC ranged from 1.10 to 2.58 mg gallic acid equivalents/g fresh weight (mg GAE/g FW) among the fleshes, from 2.80 to 4.97 mg GAE/g FW among the peels, and from 5.27 to 9.80 mg GAE/g FW among the nodes. TFC ranged from 1.89 to 6.33 mg rutin equivalents/g fresh weight (mg RE/g FW) among the fleshes, from 5.35 to 9.63 mg RE/g FW among the peels, and from 12.46 to 16.72 RE mg/g FW among the nodes. Both TPC and TFC exhibited high coefficients of variation (CV) among the lotus root varieties, ranging from 14.01% to 33.33%. “Guixi Fuou”, “Baheou”, “No. 2 Wuzhi”, “Changzhou Piaojiang” and “Baipaozi” showed relatively higher contents of phenolic compounds in comparison with the corresponding mean values. The mean value of TPC in the fleshes (1.81 mg GAE/g FW) was obviously higher than that measured by Yang (1.46 mg GAE/g FW) [6], which might be partly derived from the differences in genotype and extraction solvent. Methanol was more suited to the extraction of phenolics from lotus root than ethanol, acetone, ethyl acetate, dichloromethane, and petroleum ether [6]. The TPC of “No. 5 elian” flesh (1.39mg GAE/g FW) was close to the previous result using 80% ethanol solution as extraction solvent [6]. Phenolic compounds were widely investigated in both free and bound forms [28,29]. However, the bound phenolics of lotus root were ignored because of their low contents in all the parts. The highest contents of bound phenolics and bound flavonoids, both found in node, were 0.29 mg GAE/g FW and 0.36 mg RE/g FW, respectively [25].

### 2.2. Contents of Individual Phenolic Compounds in the Different Part of Lotus Root Varieties

On the basis of the previous investigations involved in the phenolic composition of lotus root [5,14,24,25] and other lotus tissues [19,20,21,22,23], the contents of Gallic acid, *p*-cumaric acid, gallocatechin, catechol, chlorogenic acid, (+)-catechin, caffeic acid,(−)-epicatechin and rutin were selectively determined by a high performance liquid chromatography (HPLC) method. Gallocatechin and catechin were the main phenolic compounds determined in lotus root flesh (Table 2), their mean contents were 893.57 and 18.92 µg/g FW, and their CVs of content among varieties were 25.39% and 23.36%, respectively. Gallic acid and epicatechin were detected in some of the varieties with relatively low contents. The contents of Gallic acid were in the range of 7.01–12.18 µg/g FW, and those of epicatechin were in the range of 10.87–18.73 µg/g FW. The results agreed with the previous works: the phenolic compounds of “Damaojie” flesh were mainly composed of gallic acid, d-(+)-catechin and l-(−)-epicatechin [14]; and those of “Piaohua” flesh juice were principally identified to be (+/−)-gallocatechin and (+)-catechin [24]. In addition, the flesh of “No. 2 Wuzhi” contained 8.69 µg/g FW of catechol, and that of “Baheou” contained 9.40 µg/g FW of quercetin.

The phenolic composition of peel, mainly including gallic acid, gallocatechin, catechol, catechin, caffeic acid, epicatechin and rutin, was obviously different from that of flesh, and also showed obvious differences among the varieties, as shown in Table 3. Gallic acid, gallocatechin, catechol and catechin were detected in the peels of all thirteen varieties, their mean contents were 23.80, 761.59, 6.04 and 22.66 µg/g FW, and their CVs of content among varieties were 71.32%, 50.05%, 20.81% and 26.09%, respectively. Among which, gallic acid was confirmed to be the primary substrate participating in the browning of lotus root [30]. Caffeic acid, epicatechin and rutin were detected in most of the peels individually in the content range of 1.52–32.46 µg/g FW, 29.89–151.52 µg/g FW and 15.57–46.69 µg/g FW, respectively. In addition, *p*-cumaric acid, chlorogenic acid and quercetin were not detected in peel for more than half of the varieties. Uniquely, the peel of “Baipaozi” contained 34.14 µg/g FW of *p*-cumaric acid.

The phenolic composition of node was almost the same as that of peel (Table 4). Gallic acid (mean content 26.76 µg/g FW, CV 47.99%), gallocatechin (mean content 840.44 µg/g FW, CV 40.65%), catechol (mean content 7.87µg/g FW, CV 18.75%), catechin (mean content 31.82 µg/g FW, CV 45.58%) and epicatechin (mean content 37.50 µg/g FW, CV 53.76%) were all detected in the tested nodes. Both the mean contents of gallocatechin and catechin in node were obviously higher than those in peel. The information on the phenolic composition of node was rare. Li et al. found that the hydrolysable tannins isolated from “Damaojie” node were mainly composed of gallic acid and ellagic acid [31]. Moreover, other phenolic compounds mentioned in the previous work, such asleucocyanidin and leucodephinidin [4], were not found in all the parts of lotus root. To sum up, lotus root exhibited the germplasm diversity in phenolic distribution and composition, and the diversity might be useful for resource identification and evaluation.

### 2.3. Antioxidant Activities of Phenolic Compounds from the Different Parts of Lotus Root Varieties

Oxidative stress, defined as the imbalance between free radical production and antioxidant defenses, is a condition associated with many chronic-degenerative diseases. Effects of fruits, vegetables and their antioxidants on the improvement of antioxidant levels in vivo have attracted much attention, and the resulting antioxidant activities are attributed to various mechanisms [32]. Herein, 2,2-diphenyl-1-picrylhydrazyl (DPPH) radical scavenging capacity assay and reducing antioxidant power (FRAP) assay were selected to evaluate the antioxidant activities of phenolics extracts from lotus root, as shown in Table 5. The mean concentrations for 50% inhibition (IC_50_) of DPPH radical of flesh, peel and node were 46.00, 26.43 and 21.72 µg GAE/mL, and those of FRAP antioxidant activity were 62.18, 82.05 and 95.14 µg TE/100 µg GAE, respectively. The antioxidant ability of lotus root could be characterized as node > peel > flesh, which was consistent with the previous investigations [1,6,25]. Among the thirteen varieties, the IC_50_ values of DPPH radical scavenging showed significant differences with the CV of 14.03%, 30.42% and 11.81%, respectively. The values of FRAP for peel and node, by contrast, exhibited minor differences (CV < 10%). The antioxidant activities of lotus root and its extracts showed significant correlations with TPC [1,6,33], and this correlation was widely confirmed in similar investigations on other plant materials, such as mulberry leaves [34], apples [35] and brown rice [29,36]. To explore the main components contributing to the antioxidant activity of lotus root, the Pearson′s correlation between individual phenolic contents and antioxidant activities was analyzed. According to the correlation coefficients summarized in Table 6, the two antioxidant activities had a negative correlation (*p* < 0.001); DPPH radical scavenging activity showed significant correlations with both the contents of catechin and epicatechin (*p* < 0.05); and FRAP antioxidant activity exhibited a positive correlation with catechin content (*p* < 0.001). Lachman et al. deemed that the antioxidant activity of potato flesh was synthetically provided by chlorogenic acid, gallic acid, caffeic acid and catechin [37]. The co-existence of different phenolic compounds might result in the synergistic action of antioxidant activity, which might be related to their structures [28]. Compounds with catechol moieties, multiple hydroxyl groups, and conjugation with electron-donating groups at the 4-location of the aromatic ring positively impacted the antioxidant activity [28]. It was implied that the main contributors to the antioxidant activity of lotus root might be catechin and epicatechin, but the potential synergistic effect and the relationship between structure and activity were unavailable.

**Figure 1:**
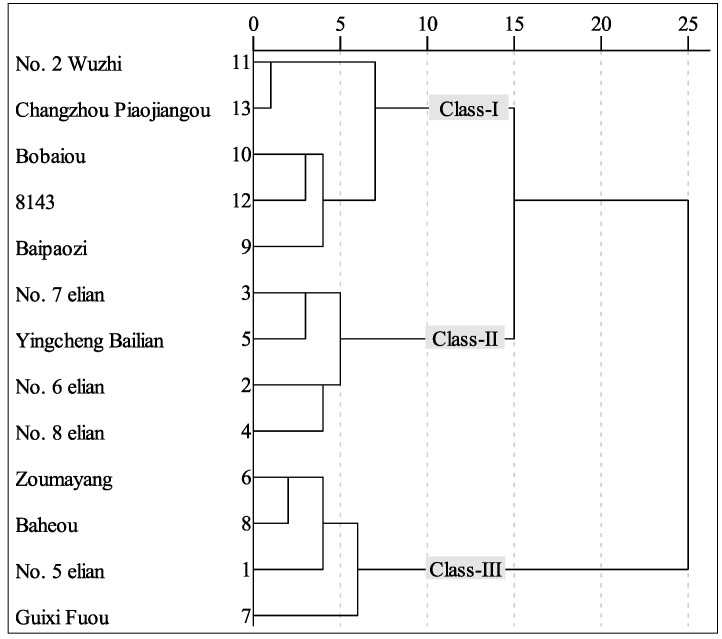
Hierarchical cluster diagram of lotus root varieties based on the content and antioxidant activity of phenolic compounds.

A hierarchical cluster analysis, based on Ward′s linkage and squared Euclidean distance using the “0–1”standardized values of TPC, TFC, DPPH radical scavenging (IC_50_) and FRAP antioxidant activity, was conducted to measure the similarity among lotus root varieties, as seen in Figure 1. The varieties fell into three classes at distance 10, in which Class-I included “No. 2 Wuzhi”, “Changzhou Piaojiangou”, “Bobaiou”, “8143”and “Baipaozi”; Class-II included “No. 7 elian”, “Yingcheng Bailian”, “No. 6 elian” and “No. 8 elian”; and Class-III included “Zoumayang”, “Baheou”, “No. 5 elian” and “Guixi Fuou”. The difference of phenolic compounds among the classes was explored (Table 7). The TPC values of flesh and node both showed no significant difference among the classes (*p* > 0.05), as well as the TFC values. Class-I and Class-III exhibited no significant difference in the TPC and TFC of peel (*p* > 0.05), and both had higher content values compared with Class-II. The antioxidant difference between Class-I and Class-II was statistically insignificant (*p* > 0.05), except that in the FRAP antioxidant activity of node (*p* < 0.05). Class-III had stronger activities than Class-I involving in the FRAP antioxidant activity of all three parts and the DPPH radical scavenging activity of node (*p* < 0.05). The activities of Class-II were comparable to those of Class-III, except the DPPH radical scavenging activity of flesh (*p* < 0.05). It was suggested that Class-III were better for developing novel value-added antioxidant products. However, none of the varieties in Class-III have been deeply investigated on the antioxidant effect of phenolic compounds.

## 3. Materials and Methods

### 3.1. Plant Material and Chemicals

Thirteen commercially cultivated varieties of lotus root, namely “No. 5 elian”, “No. 6 elian”, “No. 7 elian”, “No. 8 elian”, “Yingcheng Bailian”, “Zoumayang”, “Guixi Fuou”, “Baheou”, “Baipaozi”, “Bobaiou”, “No. 2 Wuzhi”, “8143”and “Changzhou Piaojiangou”, were provided by National Aquatic Vegetable Germplasm Repository (Wuhan, China) and identified by professor Yi-man Liu and senior agronomist Jing Peng (Wuhan Vegetable Research Institute, Wuhan, China). The fresh materials were harvested in October 2015. After cleaning well and cooling to 4 °C, lotus roots were manually cut into three parts, flesh, peel (about 0.1 cm thick) and node, followed by grinding individually in a food processor (HR7629/00, Philips Corporation, Huizhou, China). The prepared materials were then stored at −20 °C.

DPPH, TPTZ (2,4,6-tripyridyl-Striazine), Trolox (6-hydroxy-2,5,7,8-tetramethylchroman-2-carboxylic acid), gallic acid and rutin were purchased from Tokyo Chemical Industry Co., Ltd (Shanghai, China). Folin-Ciocalteu, (+)-catechin, (−)-epicatechin, resveratrol, *p*-cumaric acid, caffeic acid, quercetin and chlorogenic acid were purchased from Sigma-Aldrich Co., LLC (St. Louis, MO, USA). HPLC-grade acetonitrile and acetic acid were purchased from Fisher Scientific Co. (Pittsburgh, PA, USA). Other chemicals were analytical grade and purchased from Sinopharm Chemical Reagent Co., Ltd. (Shanghai, China).

### 3.2. Extraction of Phenolic Compounds

The phenolic compounds were extracted from different parts of lotus root according to previous methods [25,29] with minor modifications. Flesh, peel or node samples (5.0 g), soaked in 50 mL of 4 °C pre-cooled 80% methanol acidified with 2% formic acid, were homogenized at 10,500 r/min for 4 min using a XHF-D high-shear homogenizer (Ningbo xinzhi biotechnology Co., Ltd, Ningbo, China). The homogenate was centrifuged at 10,000 r/min for 5 min, followed by filtration through a Whatman No. 1 filter paper, to separate supernatant. The residues were homogenized with 50 mL solvent again to collect supernatant. The supernatants were combined and concentrated at 50 °C using a vacuum rotary evaporator (BC-R203, Shanghai Biochemical Equipment Co., Shanghai, China), and diluted with methanol to 25 mL. The extract was then filtered through a 0.45-μm syringe filter prior to subsequent analysis. The preparation was performed in triplicate for each sample.

### 3.3. Determination of Total Phenolics and Total Flavonoids

TPC was measured according to the Folin-Ciocalteu method [38] with minor modifications. The phenolic extract (0.125 mL), Folin-Ciocalteu reagent (0.125 mL) and distilled water (0.5 mL) were mixed in a centrifuge tube. After incubation for 6 min, 2.25 mL of 3.5% Na_2_CO_3_ solution (*m*/*v*) was added. The mixture was then kept in dark at room temperature for 90 min, followed by absorbance determination at 760 nm using a UV-visible spectrophotometer (Shimadzu, UV-1800, Kyoto, Japan). Meanwhile, gallic acid (50–500 μg/mL) was used as standard to establish a calibration curve. TPC (mg GAE/g FW) was measured in triplicate.

TFC was measured according to the method described by Jia et al. [39], with minor modifications. Briefly, 0.3 mL phenolic extract was sequentially mixed with 1.5 mL methanol, 0.09 mL of 5% NaNO_2_ solution (*m*/*v*), and 0.18 mL of 10% AlCl_3_∙6H_2_O solution (*m*/*v*). After incubation for 5 min, 0.6 mL of 1 mol/L NaOH solution and 0.33 mL distilled water were added to the mixture. The absorbance of the mixture was read at 510 nm. Meanwhile, rutin (20–100 μg/mL) was used as standard to establish a calibration curve. TFC (mg RE/g FW) was measured in triplicate.

### 3.4. Chromatographic Analysis of Phenolic Compounds

HPLC analysis was conducted on a Waters series system equipped with a 2487 ultraviolet-visible detector, a 1525 binary pump (Waters, Milford, MA, USA) and a Luna C18(2) column (250 mm × 4.6 mm, Phenomenex, Torrance, CA, USA) maintained at 30 °C. The flow rate was 1.0 mL/min, the injection volume was 20 μL, and the absorbance was detected at 280 nm. The mobile phases consisted of (A) acetonitrile and (B) 0.4% acetic acid (*v*/*v*). An elution gradient was implemented as follows: 5% (A) and 95% (B) to 25% (A) and 75% (B) over 30 min; to 50% (A) and 50% (B) over 10 min; to 5% (A) and 95% (B) over 15 min; and re-equilibration over 10 min. External standard method and internal standard method were simultaneously used for the identification of chromatographic peaks with authentic standards. Each sample was performed in triplicate. The calibration curve of each standard, based on serial concentrations and their corresponding peak areas, was used for quantitation.

### 3.5. Evaluation of Antioxidant Activities

DPPH radical scavenging activity was evaluated by the method of Brand-Williams et al. [40], with slight modifications. Briefly, 50 μL sample solution with an appropriate phenolic concentration adjusted by methanol was mixed with 0.7 mL of a 0.1 mol/mL methanolic solution of DPPH. After incubation under darkness at room temperature for 30 min, the absorbance of the mixture was measured at 517 nm. All determinations were performed in triplicate. The scavenging percent (S, %) of DPPH radicals was calculated using the equation:

S (%) = ((A_c_ − A_s_)/A_c_) × 100 (1)

where A_c_ is the absorbance of the control, and A_s_ is the absorbance of the sample. IC_50_ value was calculated as the phenolic concentration (µg GAE/mL) with 50% scavenging of DPPH radicals.

Total antioxidant power was determined using the FRAP assay [41], with slight modifications. The FRAP reagent was prepared by mixing 300 mmol/L acetate buffer (pH 3.6), 10 mmol/L TPTZ solution in 40 mmol/L HCl and 20 mmol/L FeCl_3_ solution at a volume ratio of 10:1:1, and preheated to 37 °C for use. Sample solution (1 mL) with an appropriate phenolic concentration adjusted by methanol was mixed with 1.8 mL FRAP reagent. After incubation under darkness at room temperature for 10 min, the absorbance of the mixture was measured at 593 nm. All determinations were performed in triplicate. Different concentrations of Trolox were determined for a standard curve. The FRAP antioxidant activity of sample solution was corrected with total phenolics concentration and expressed as µg TE/100 µg GAE.

### 3.6. Statistical Analysis

IBM SPSS Statistics 19 software (IBM, Armonk, NY, USA) was used for statistical analysis. The data were expressed as means ± standard deviations from triplicate experiments. The significance of difference at a level of 0.05 was evaluated with ANOVA (One-way analysis of variance) and SNK (Student-Newman-Keuls) test. Pearson’s correlation test was used to identify correlation between groups. Hierarchical cluster analysis was conducted with Ward´s method of linkage and squared Euclidean distance as a measure of the similarity among varieties.

## 4. Conclusions

Thirteen extensively cultivated varieties of lotus root were investigated on phenolic profiles and antioxidant activity for a better understanding on the high-value utilization of this aquatic vegetable in functional food industry. The varieties showed significant differences in the content, distribution, composition and antioxidant activity of phenolic compounds. “Zoumayang”, “Baheou”, “No. 5 elian” and “Guixi Fuou” possessed relatively higher phenolic content and stronger antioxidant activity compared with others. As the main by-products of lotus root, node and peel exhibited higher phenolics content, stronger antioxidant activity and more phenolic compounds than the edible flesh. The differences among the varieties and parts should be considered for consumption and nutraceutical purposes. Catechin and epicatechin were suggested to be the primary contributors to the antioxidant activity of lotus root, but the potential synergies cooperated with other phenolic compounds should be further explored. Therefore, the fingerprint of phenolic compounds of lotus root needs to be further analyzed in detail.

## Figures and Tables

**Table 1:** Contents of total phenolics and total flavonoids in the different parts of lotus root varieties.

Varieties	Total Phenolics Content (mg GAE/g FW)			Total Flavonoids Content (mg RE/g FW)		
	Flesh	Peel	Node	Flesh	Peel	Node
No. 5 elian	1.39 ± 0.05 ^b^	4.97 ± 0.04 ^h^	6.09 ± 0.06 ^b^	2.40 ± 0.13 ^b^	9.26 ± 0.00 ^f^	12.46 ± 0.72 ^a^
No. 6 elian	1.44 ± 0.08 ^b^	2.80 ± 0.16 ^a^	6.94 ± 0.12 ^c^	2.49 ± 0.09 ^b^	5.35 ± 0.09 ^a^	15.68 ± 0.28 ^cd^
No. 7 elian	1.81 ± 0.04 ^d^	3.96 ± 0.11 ^cd^	7.57 ± 0.01 ^d^	3.02 ± 0.11 ^cd^	7.03 ± 0.00 ^bc^	16.12 ± 0.97 ^d^
No. 8 elian	1.10 ± 0.06 ^a^	3.69 ± 0.16 ^b^	5.27 ± 0.04 ^a^	1.89 ± 0.10 ^a^	6.77 ± 0.17 ^b^	13.98 ± 0.06 ^b^
Yingcheng Bailian	1.69 ± 0.03 ^c^	3.89 ± 0.06 ^c^	6.95 ± 0.19 ^c^	3.58 ± 0.03 ^e^	7.38 ± 0.23 ^cde^	14.30 ± 0.95 ^bc^
Zoumayang	1.90 ± 0.04 ^de^	4.59 ± 0.07 ^e^	6.95 ± 0.17 ^c^	3.30 ± 0.17 ^de^	7.55 ± 0.20 ^e^	14.33 ± 0.10 ^bc^
Guixi Fuou	2.33 ± 0.07 ^g^	4.69 ± 0.15 ^fg^	8.14 ± 0.07 ^e^	6.33 ± 0.19 ^g^	9.63 ± 0.12 ^g^	16.72 ± 0.90 ^d^
Baheou	2.52 ± 0.05 ^h^	4.68 ± 0.07 ^fg^	6.95 ± 0.08 ^c^	4.68 ± 0.02 ^f^	9.11 ± 0.14 ^f^	14.42 ± 0.28 ^bc^
Baipaozi	1.82 ± 0.05 ^d^	4.68 ± 0.10 ^fg^	6.27 ± 0.15 ^b^	3.32 ± 0.13 ^de^	9.25 ± 0.21 ^f^	15.82 ± 0.20 ^cd^
Bobaiou	1.84 ± 0.04 ^d^	4.29 ± 0.11 ^e^	6.85 ± 0.16 ^c^	3.06 ± 0.14 ^cd^	7.14 ± 0.20 ^bcd^	13.29 ± 0.31 ^ab^
No. 2 Wuzhi	1.96 ± 0.09 ^ef^	4.92 ± 0.17 ^h^	9.80 ± 0.25 ^g^	3.51 ± 0.18 ^e^	7.47 ± 0.26 ^de^	19.34 ± 0.51 ^e^
8143	2.02 ± 0.02 ^f^	4.14 ± 0.13 ^de^	8.31 ± 0.10 ^e^	2.91 ± 0.11 ^c^	6.69 ± 0.11 ^bc^	16.11 ± 0.87 ^d^
Changzhou Piaojiangou	1.66 ± 0.01 ^c^	4.54 ± 0.10 ^f^	9.44 ± 0.16 ^f^	3.07 ± 0.09 ^cd^	7.36 ± 0.15 ^cde^	19.98 ± 0.93 ^e^
Mean	1.81	4.30	7.35	3.35	7.69	15.58
Coefficient of variation	20.87%	14.01%	17.54%	33.33%	16.36%	14.05%

The statistical differences in individual content among varieties were evaluated with One-way analysis of variance and Student–Newman–Keuls test. Data marked with different letters are significantly different (*p* < 0.05), and marked with same letter are statistically indifferent (*p* > 0.05).

**Table 2:** Contents of individual phenolic compounds in the fleshes of lotus root varieties.

Varieties	Content of Phenolic Compounds (µg/g FW)			
	Gallic Acid	Gallocatechin	Catechin	Epicatechin
No. 5 elian	-	475.22 ± 8.98	13.51 ± 0.38	11.94 ± 0.39
No. 6 elian	12.18 ± 1.10	766.82 ± 26.65	18.56 ± 0.65	14.18 ± 1.05
No. 7 elian	-	536.42 ± 30.99	20.12 ± 1.09	-
No. 8 elian	9.80 ± 1.52	770.62 ± 3.01	11.07 ± 0.22	18.73 ± 0.13
Yingcheng Bailian	-	996.25 ± 94.77	24.49 ± 4.33	-
Zoumayang	7.01 ± 0.35	896.18 ± 10.91	19.71 ± 0.18	10.87 ± 1.97
Guixi Fuou	-	1184.79 ± 21.33	23.64 ± 0.11	11.26 ± 0.23
Baheou	8.82 ± 0.70	1150.82 ± 12.95	25.02 ± 0.23	11.94 ± 0.39
Baipaozi	-	1133.45 ± 12.63	17.98 ± 0.41	12.03 ± 0.74
Bobaiou	-	856.09 ± 9.93	13.09 ± 0.37	14.30 ± 0.76
No. 2 Wuzhi	-	923.70 ± 43.71	22.01 ± 0.77	-
8143	-	1125.76 ± 42.30	20.08 ± 0.94	-
Changzhou Piaojiangou	-	800.25 ± 5.66	16.67 ± 0.68	-

- means the compound has not been detected.

**Table 3:** Contents of individual phenolic compounds in the peels of lotus root varieties.

Varieties	Content of Phenolic Compounds (µg/g FW)									
	Gallic Acid	*p*-Cumaric Acid	Gallocatechin	Catechol	Chlorogenic Acid	Catechin	Caffeic Acid	Epicatechin	Rutin	Quercetin
No. 5 elian	29.67 ± 2.61	13.79 ± 1.85	282.45 ± 16.53	7.09 ± 0.46	-	21.55 ± 1.37	5.91 ± 0.36	131.17 ± 9.51	34.46 ± 1.91	-
No. 6 elian	53.72 ± 8.64	-	280.74 ± 3.67	4.10 ± 0.03	-	12.48 ± 0.34	6.75 ± 0.30	151.52 ± 15.35	15.57 ± 1.32	-
No. 7 elian	51.14 ± 1.32	-	379.02 ± 17.22	5.36 ± 0.25	-	22.61 ± 2.01	6.10 ± 1.09	49.68 ± 4.11	23.52 ± 2.34	-
No. 8 elian	37.97 ± 3.89	-	494.63 ± 5.52	5.30 ± 0.07	-	20.98 ± 0.18	2.96 ± 0.27	61.41 ± 3.81	46.69 ± 3.79	146.77 ± 11.09
Yingcheng Bailian	23.25 ± 1.84	-	742.34 ± 68.92	6.42 ± 0.11	-	27.87 ± 1.49	-	29.89 ± 1.06	26.14 ± 1.81	9.70 ± 0.39
Zoumayang	26.60 ± 3.44	86.51 ± 3.08	1113.40 ± 123.55	4.20 ± 0.37	40.59 ± 3.99	16.08 ± 0.61	5.65 ± 0.37	106.84 ± 6.01	18.56 ± 1.43	-
Guixi Fuou	4.36 ± 0.60	-	1583.30 ± 128.74	8.16 ± 1.26	-	30.87 ± 3.31	2.86 ± 0.26	65.20 ± 3.28	22.03 ± 0.79	-
Baheou	24.49 ± 1.69	42.50 ± 2.46	975.24 ± 119.89	6.76 ± 0.32	46.26 ± 4.82	12.72 ± 0.75	3.90 ± 0.12	63.60 ± 0.81	24.52 ± 2.30	10.62 ± 0.16
Baipaozi	5.37 ± 0.09	-	1088.20 ± 339.46	7.28 ± 0.92	36.27 ± 0.30	22.46 ± 3.72	4.75 ± 0.49	109.88 ± 9.67	23.15 ± 3.17	-
Bobaiou	5.12 ± 0.25	47.32 ± 0.11	747.94 ± 51.31	5.12 ± 0.05	-	28.69 ± 1.51	3.70 ± 0.11	66.50 ± 2.52	-	9.94 ± 0.10
No. 2 Wuzhi	13.13 ± 1.54	-	747.00 ± 21.21	6.66 ± 0.46	-	26.62 ± 0.99	32.46 ± 0.86	-	31.65 ± 1.74	-
8143	29.74 ± 2.43	-	969.20 ± 111.19	5.61 ± 0.17	-	23.51 ± 2.46	1.52 ± 0.05	38.56 ± 3.13	-	-
Changzhou Piaojiangou	4.91 ± 0.19	11.21 ± 0.70	496.93 ± 21.28	7.52 ± 0.26	-	27.06 ± 1.04	2.63 ± 0.08	88.96 ± 1.15	23.73 ± 0.87	-

- means the compound has not been detected.

**Table 4:** Contents of individual phenolic compounds in the nodes of lotus root varieties.

Varieties	Content of Phenolic Compounds (µg/g FW)									
	Gallic Acid	*p*-Cumaric Acid	Gallocatechin	Catechol	Chlorogenic Acid	Catechin	Caffeic Acid	Epicatechin	Rutin	Quercetin
No. 5 elian	23.07 ± 2.26	14.55 ± 1.65	424.64 ± 15.80	6.51 ± 0.57	-	37.78 ± 2.99	1.91 ± 0.20	34.40 ± 0.66	-	-
No. 6 elian	37.98 ± 1.27	12.04 ± 0.16	527.43 ± 2.50	7.50 ± 0.15	-	37.55 ± 5.36	2.87 ± 0.51	40.14 ± 0.83	10.43 ± 0.50	-
No. 7 elian	34.10 ± 2.82	-	436.25 ± 26.90	6.82 ± 0.88	-	25.63 ± 2.38	7.66 ± 1.10	18.52 ± 1.45	-	-
No. 8 elian	28.86 ± 3.69	-	612.35 ± 53.73	8.25 ± 0.54	-	33.89 ± 1.07	-	18.26 ± 2.00	18.05 ± 2.16	-
Yingcheng Bailian	19.72 ± 0.30	72.72 ± 3.10	1112.38 ± 23.80	10.36 ± 0.08	-	61.15 ± 1.22	-	14.69 ± 1.79	25.41 ± 1.25	-
Zoumayang	38.36 ± 2.55	-	979.08 ± 18.18	6.40 ± 0.49	47.67 ± 0.66	13.84 ± 0.99	1.90 ± 0.18	29.22 ± 3.51	19.60 ± 2.17	-
Guixi Fuou	48.27 ± 3.94	-	1257.40 ± 30.15	9.33 ± 0.47	-	12.17 ± 0.60	4.28 ± 0.48	82.89 ± 3.29	29.36 ± 1.35	9.22 ± 0.16
Baheou	15.50 ± 0.74	32.54 ± 2.95	883.07 ± 23.84	7.72 ± 0.59	55.34 ± 5.36	42.32 ± 4.19	1.96 ± 0.01	18.99 ± 0.30	-	9.54 ± 0.04
Baipaozi	30.28 ± 2.26	-	1411.69 ± 164.69	6.22 ± 0.54	-	15.18 ± 2.40	3.81 ± 1.39	38.10 ± 2.69	-	11.44 ± 1.11
Bobaiou	5.26 ± 2.35	36.02 ± 2.50	340.32 ± 29.30	8.38 ± 0.86	-	35.60 ± 3.84	5.24 ± 0.47	48.88 ± 1.14	-	10.26 ± 0.21
No. 2 Wuzhi	39.65 ± 6.05	-	1028.34 ± 45.42	5.89 ± 0.19	45.30 ± 1.06	15.74 ± 1.99	6.44 ± 0.35	43.91 ± 0.85	28.56 ± 1.93	-
8143	15.66 ± 2.82	47.76 ± 4.70	897.09 ± 36.42	9.15 ± 0.70	-	41.56 ± 2.86	3.75 ± 0.54	31.46 ± 2.78	22.00 ± 0.77	22.90 ± 4.18
Changzhou Piaojiangou	11.16 ± 2.65	64.51 ± 3.94	1015.70 ± 39.24	9.84 ± 0.76	-	41.20 ± 2.73	5.24 ± 0.52	68.01 ± 7.70	-	-

- means the compound has not been detected.

**Table 5:** Antioxidant activities of phenolic compounds from the different parts of lotus root varieties.

Varieties	IC_50_ of DPPH Radical Scavenging (µg GAE/mL)			FRAP Antioxidant Activity (µg TE/100 µg GAE)		
	Flesh	Peel	Node	Flesh	Peel	Node
No. 5 elian	37.44 ± 1.52 ^a^	22.37 ± 1.06 ^ab^	19.04 ± 0.37 ^a^	79.92 ± 7.31 ^bc^	92.59 ± 3.78 ^b^	110.08 ± 3.69 ^cd^
No. 6 elian	59.39 ± 1.25 ^e^	24.80 ± 2.71^ab^	18.98 ± 1.38 ^a^	75.23 ± 2.64 ^b^	82.88 ± 2.72 ^ab^	105.70 ± 5.81 ^bcd^
No. 7 elian	49.99 ± 0.74 ^c^	24.11 ± 0.94 ^ab^	24.07 ± 0.85 ^b^	86.16 ± 1.44 ^cd^	84.64 ± 3.84 ^ab^	106.97 ± 2.80 ^bcd^
No. 8 elian	43.36 ± 1.21 ^b^	23.78 ± 3.08 ^ab^	21.05 ± 0.02 ^a^	63.13 ± 1.62 ^a^	94.15 ± 3.45 ^b^	107.20 ± 7.31 ^bcd^
Yingcheng Bailian	55.04 ± 3.96 ^d^	51.50 ± 2.05 ^d^	21.14 ± 1.83 ^a^	77.71 ± 2.88 ^b^	92.17 ± 2.87 ^b^	107.29 ± 10.46 ^bcd^
Zoumayang	43.46 ± 2.58 ^b^	22.95 ± 1.38 ^ab^	18.74 ± 0.41 ^a^	94.38 ± 1.92 ^e^	98.15 ± 9.50 ^b^	98.74 ± 2.99 ^bcd^
Guixi Fuou	42.73 ± 3.49 ^b^	19.94 ± 0.83 ^a^	19.96 ± 0.65 ^a^	74.10 ± 5.81 ^b^	94.32 ± 5.10 ^b^	112.32 ± 2.11 ^d^
Baheou	37.52 ± 3.13 ^a^	20.23 ± 1.29 ^a^	19.66 ± 0.18 ^a^	91.00 ± 4.32 ^de^	93.28 ± 6.73 ^b^	100.59 ± 9.96 ^bcd^
Baipaozi	49.55 ± 1.45 ^c^	25.28 ± 3.62 ^ab^	24.90 ± 1.64 ^b^	76.12 ± 5.31 ^b^	87.35 ± 2.25 ^ab^	78.78 ± 2.38 ^a^
Bobaiou	43.06 ± 0.91 ^b^	24.61 ± 2.67 ^ab^	20.42 ± 0.84 ^a^	65.38 ± 6.05 ^a^	82.41 ± 4.17 ^ab^	92.76 ± 8.37 ^b^
No. 2 Wuzhi	42.67 ± 1.94 ^b^	25.86 ± 1.19 ^b^	24.23 ± 0.70 ^b^	75.65 ± 1.82 ^b^	82.20 ± 4.85 ^ab^	97.19 ± 1.93 ^bcd^
8143	50.03 ± 2.01 ^c^	30.45 ± 1.53 ^c^	24.54 ± 0.26 ^b^	65.83 ± 3.06 ^a^	73.33 ± 5.86 ^a^	92.82 ± 3.01 ^b^
Changzhou Piaojiangou	43.75 ± 2.79 ^b^	27.70 ± 1.39 ^bc^	25.68 ± 1.34 ^b^	62.18 ± 2.34 ^a^	82.05 ± 7.47 ^ab^	95.14 ± 4.61 ^bc^
Mean	46.00	26.43	21.72	75.91	87.66	100.43
Coefficient of variation	14.03%	30.42%	11.81%	13.51%	8.06%	9.20%

The statistical difference in individual activity among varieties was evaluated with One-way analysis of variance and Student-Newman-Keuls test. Data marked with different letters are significantly different (*p* < 0.05), and marked with same letter are statistically indifferent (*p* > 0.05).

**Table 6:** Pearson′s correlation between phenolic contents and antioxidant activities.

Variates	Coefficient of Correlation	
	IC_50_ of DPPH Radical Scavenging	FRAP Antioxidant Activity
IC_50_ of DPPH radical scavenging (*n* = 39)		−0.672 **
FRAP antioxidant activity (*n* = 39)	−0.672 **	
Gallic acid content (*n* = 30)	−0.308	0.274
Gallocatechin content (*n* = 39)	0.104	−0.088
Catechol content (*n* = 27)	−0.066	0.358
Catechin content (*n* = 39)	−0.332 *	0.473 **
Epicatechin content (*n* = 34)	−0.433 *	0.103

** means the significance level of *p*< 0.001, and * means the significance level of *p* < 0.05.

**Table 7:** Phenolic contents and antioxidant activities of three classes of lotus root.

Classification		Class-I	Class-II	Class-III
Flesh	TPC (mg GAE/g FW)	1.86 ± 0.14 ^a^	1.51 ± 0.31 ^a^	2.04 ± 0.50 ^a^
	TFC (mg RE/g FW)	3.17 ± 0.24 ^a^	2.74 ± 0.72 ^a^	4.18 ± 1.71 ^a^
	IC_50_ of DPPH radical scavenging (µg GAE/mL)	45.81 ± 3.66 ^ab^	51.94 ± 6.89 ^b^	40.29 ± 3.26 ^a^
	FRAP antioxidant activity (µg TE/100 µg GAE)	69.03 ± 6.41 ^a^	75.56 ± 9.51 ^ab^	84.85 ± 9.46 ^b^
Peel	TPC (mg GAE/g FW)	4.51 ± 0.31 ^b^	3.58 ± 0.54 ^a^	4.73 ± 0.16 ^b^
	TFC (mg RE/g FW)	7.58 ± 0.98 ^ab^	6.63 ± 0.89 ^a^	8.89 ± 0.92 ^b^
	IC_50_ of DPPH radical scavenging (µg GAE/mL)	26.78 ± 2.35 ^a^	31.05 ± 13.64 ^a^	21.37 ± 1.51 ^a^
	FRAP antioxidant activity (µg TE/100 µg GAE)	81.47 ± 5.06 ^a^	88.46 ± 5.53 ^ab^	94.58 ± 2.48 ^b^
Node	TPC (mg GAE/g FW)	8.14 ± 1.55 ^a^	6.68 ± 0.99 ^a^	7.03 ± 0.84 ^a^
	TFC (mg RE/g FW)	16.91 ± 2.75 ^a^	15.02 ± 1.04 ^a^	14.48 ± 1.74 ^a^
	IC_50_ of DPPH radical scavenging (µg GAE/mL)	23.95 ± 2.05 ^b^	21.31 ± 2.09 ^ab^	19.35 ± 0.56 ^a^
	FRAP antioxidant activity (µg TE/100 µg GAE)	91.34 ± 7.25 ^a^	106.79 ± 0.74 ^b^	105.43 ± 6.76 ^b^

The statistical differences among classes were evaluated with One-way analysis of variance and Student-Newman-Keuls test. Data marked with different letters are significantly different (*p* < 0.05), and marked with same letter are statistically indifferent (*p* > 0.05).
